# Senescence-accelerated mice prone 8 (SAMP8) in male as a spontaneous osteoarthritis model

**DOI:** 10.1186/s13075-022-02916-5

**Published:** 2022-10-18

**Authors:** Yohei Sanada, Yasunari Ikuta, Chenyang Ding, Masahiro Shinohara, Dilimulati Yimiti, Hiroyuki Ishitobi, Keita Nagira, Minjung Lee, Takayuki Akimoto, Sachi Shibata, Masakazu Ishikawa, Tomoyuki Nakasa, Kiminori Matsubara, Martin K. Lotz, Nobuo Adachi, Shigeru Miyaki

**Affiliations:** 1 Medical Center for Translational and Clinical Research, Hiroshima University Hospital, 1-2-3 Kasumi Minami-ku, Hiroshima, 734-8551 Japan; 2 Department of Orthopaedic Surgery, Graduate School of Biomedical and Health Sciences, Hiroshima University, Hiroshima, Japan; 3 Department of Rehabilitation for the Movement Functions, National Rehabilitation Center for Persons With Disabilities, Saitama, Japan; 4 Department of Orthopaedic Surgery, Tottori University, Tottori, Japan; 5 Faculty of Sport Sciences, Waseda University, Saitama, Japan; 6 Department of Human Life Science Education, Graduate School of Education, Hiroshima University, Higashi-Hiroshima, Japan; 7 Department of Molecular Medicine, Scripps Research, La Jolla, San Diego, CA USA

**Keywords:** Osteoarthritis, Aging, Mouse models, Subchondral bone, Meniscus

## Abstract

**Background:**

Animal models of spontaneous osteoarthritis (OA) are sparse and not well characterized. The purpose of the present study is to examine OA-related changes and mechanisms in senescence-accelerated mouse prone 8 (SAMP8) that displays a phenotype of accelerated aging.

**Methods:**

Knees of male SAMP8 and SAM-resistant 1 (SAMR1) mice as control from 6 to 33 weeks of age were evaluated by histological grading systems for joint tissues (cartilage, meniscus, synovium, and subchondral bone), and µCT analysis. Gene expression patterns in articular cartilage were analyzed by real-time PCR. Immunohistochemistry was performed for OA-related factors, senescence markers, and apoptosis.

**Results:**

Starting at 14 weeks of age, SAMP8 exhibited mild OA-like changes such as proteoglycan loss and cartilage fibrillation. From 18 to 33 weeks of age, SAMP8 progressed to partial or full-thickness defects with exposure of subchondral bone on the medial tibia and exhibited synovitis. Histological scoring indicated significantly more severe OA in SAMP8 compared with SAMR1 from 14 weeks [median (interquartile range): SAMR1: 0.89 (0.56–1.81) vs SAMP8: 1.78 (1.35–4.62)] to 33 weeks of age [SAMR1: 1.67 (1.61–1.04) vs SAMP8: 13.03 (12.26–13.57)]. Subchondral bone sclerosis in the medial tibia, bone mineral density (BMD) loss of femoral metaphysis, and meniscus degeneration occurred much earlier than the onset of cartilage degeneration in SAMP8 at 14 weeks of age.

**Conclusions:**

SAMP8 are a spontaneous OA model that is useful for investigating the pathogenesis of primary OA and evaluating therapeutic interventions.

**Supplementary Information:**

The online version contains supplementary material available at 10.1186/s13075-022-02916-5.

## Introduction

Osteoarthritis (OA), the most common joint disease, is characterized by joint pain and decreased joint function and caused by various risk factors such as aging, traumatic injury, and obesity. The disease process is associated with changes in all joint tissues, including cartilage, subchondral bone, trabecular bone, synovium, menisci, and ligaments [1]. The mechanisms that mediate the effect of age on OA have not been elucidated completely. Thus, pharmacological approaches for OA prevention or disease-modification are presently not available. Although most in vivo OA studies use secondary OA model via injury, such as destabilization of the medial meniscus (DMM) with transection of ligaments [2], aging is one of the most important OA risk factors [1]. These models are useful for studying post-traumatic OA but may not be a valid approach for studying mechanisms and treatments of spontaneous aging-related OA. Thus, the understanding of primary OA pathogenesis requires animal models with spontaneous OA-like changes, which are biochemically and histologically similar to human OA.

Spontaneous OA in mice develops much more slowly than in induced OA models. In C57BL/6 J OA-prone mice, OA-like changes only become detectable at 12–18 months [3–6]. Thus far, the STR/ort mouse have been established as the only model of primary or spontaneous OA in mice [7, 8]. These mice develop severe lesions occurring by about 15 months in the majority of animals [8]. Senescence-accelerated mice (SAM) are derived from AKR/J breeding colonies, and many sublines of SAM-prone (SAMP) and SAM-resistant (SAMR) mice were developed by selective breeding [9, 10]. SAMP show characteristics of rapid aging and reduced lifespan with a relatively strain-specific phenotype. Among the strains, SAMP8 exhibits aging-related diseases such as neurodegenerative disorders including age-related deteriorations in memory and learning ability, and sarcopenia [10, 11]. However, while other and our groups reported OA-like changes including subchondral bone sclerosis in SAMP8 [12, 13], details about the temporal development of OA, and the mechanisms of OA and the suitability of SAMP8 for preclinical drug testing have not been investigated.

The purpose of the present study is to examine OA-related changes and mechanisms in SAMP8 that displays a phenotype of accelerated aging.

## Materials and methods

### Senescence-accelerated mice (SAM)

SAMP8 and SAMR1 at 3 weeks of age were obtained from Japan SLC (Shizuoka, Japan), and only male mice were used in this study as only male mice are commercially available in Japan. A total of 115 SAMR1 and 115 SAMP8 were obtained from the vendor. For the aging study, a total of 90 mice each for SAMR1 and SAMP8 were evaluated in histopathological assessments (SAMR1 *n* = 10; SAMP8 *n* = 10 at 6, 9, 11, 14, 18, 28, and 33 weeks of age; SAMR1 *n* = 20; SAMP8 *n* = 20 at 23 weeks of age). Furthermore, SAMR1 and SAMP8 at 4 weeks of age (*n* = 15 for each strain) were used for in vitro studies. All mice were housed in groups of three to five per cage (S 143 mm × 293 mm × H148mm) with a sterilized beta-chip bedding and maintained at 23 ± 1 °C with a 12-h light/dark cycle and acidified water and complete commercial pelleted food ad libitum. All animal experiments were performed according to protocols approved by the Hiroshima University Animal Care and Use Committee.

### Histopathological assessments

Knee, hip, and ankle joints from each mouse were embedded intact in paraffin after fixation in 4% paraformaldehyde phosphate buffer solution (PBS) for 48 h at 4 °C and decalcification in K-CX (FALMA, Japan) for 6 h at room temperature. Paraffin-embedded knee joints were sectioned at 4.5 μm in the coronal plane through the central weight-bearing region of the anterior and posterior femorotibial joint. Hip and ankle joints were sectioned in the sagittal plane. Three different sections per joint were stained with Safranin O (MUTO PURE CHEMICALS, Tokyo, Japan) and Fast Green (Sigma-Aldrich, USA). Histological assessments were performed on each section, and the average scores from three different sections were used for statistical analysis. Three different researchers were blinded while performing all scorings. In this study, we applied separate scoring systems for articular cartilage, meniscus, subchondral bone, and synovium. Damage of articular cartilage (maximum 24 points per knee joint section; 6 points for each quadrant of the medial and lateral femoral/tibial cartilage) was evaluated using the OARSI scoring system [14]. The criteria for determining OA severity were decided by highest score the four quadrants can reach (Mild OA: 0.5; Moderate OA: 1 to 3; Severe OA: 4 to 6). Subchondral bone changes, meniscus degradation, and the severity of synovitis were evaluated according to previously described histopathological scoring systems [13, 15, 16] using the left knee joints.

### Microcomputed tomography (µCT) analysis

Mouse lower limb was collected from SAMR1 and SAMP8 at 4, 6, 9, and 23 weeks of age and was kept in 70% EtOH after fixation in 4% paraformaldehyde phosphate buffer solution (PBS) for 48 h at 4 °C. Analysis by µCT was performed in the epiphysis of the tibias and the metaphysis of the distal femur as we previously described [13].

### Cytokine analysis in serum

Mouse serum was collected from abdominal vena cava in SAMR1 (*n* = 4) and SAMP8 (*n* = 4) at 9 weeks of age. Measurement of IL-6 in serum was carried out using Milliplex MAP technology. Multiplex Assay kit (MILLIPLEX, Merk Millipore, USA) was obtained, and the procedure was performed following the manufacturer’s instruction.

### Immunohistochemical analysis

Slides were pretreated with antigen-retrieval reagent (Immunoactive; Matsunami Glass Ind, Osaka, Japan) at 60 °C for 16 h, followed by blocking with 10% normal horse serum for 30 min. Then, sections were immunostained with anti-P16^INK4a^ antibody (abcam, ab54210; 0.1 µg/mL), anti-ADAMTS-5 antibody (GeneTex, GTX100332, 10 µg/mL), and anti-MMP13 antibody (Thermo Fisher Scientific, MA5-14,328, 20 µg/ml) diluted in Can Get Signal immunostaining solution (TOYOBO, Tokyo, Japan) using Vectastain ABC-AP alkaline phosphatase kit and AP substrate kit (Vector Laboratories, Burlingame, CA, USA) according to the manufacturers’ instructions. For type X Collagen staining, slides were pretreated with antigen-retrieval reagent (Proteinase K, Dako, CA, USA) at room temperature for 10 min, blocking serum for 30 min. Then, sections were immunostained with anti-type X Collagen antibody (DSHB, X-AC9, 5 µg/mL) diluted in PBS using VECTASTAIN Elite ABC-HRP kit and DAB substrate kit. Mouse IgG1 kappa (Thermo Fisher Scientific, 14–4714-82), mouse IgG2b kappa (14–4731-82), and rabbit IgG (14–4616-82) were used as isotype control antibodies for negative controls (Fig. S5).

### TUNEL staining

TUNEL staining was completed using an in situ detection kit for programmed cell death detection (MEBSTAIN apoptosis TUNEL Kit direct: MBL, USA) according to the manufacturer’s instructions. Nuclei were stained by 4’,6-diamidino-2-phenylindole (DAPI).

### Isolation of mouse articular cartilage and culture of mouse articular chondrocytes

Articular cartilage tissues for gene expression analysis were isolated from knee joints of mice at 4 weeks of age under a microscope. For chondrocyte isolation, cartilage was resected from the femoral heads of SAMR1 and SAMP8 at 4 weeks of age and digested with 3.5 mg/ml collagenase Type 2 (Worthington, Lakewood, NJ, USA) in Dulbecco’s modified Eagle’s medium (DMEM) (FUJIFILM Wako, Japan) for 1.5 h at 37 °C. Primary chondrocytes were seeded on a 12-well plate with DMEM with 10% fetal bovine serum and 1% penicillin/streptomycin and cultured for 7–10 days. Then, the chondrocytes (passage 1) with or without IL-1β (1 ng/ml, 24 h) were used in the present experiments.

### Quantitative real-time PCR

Total RNA was extracted from articular cartilage tissues and cultured chondrocytes using Isogen reagent (Nippon gene, Tokyo, Japan) and RNA purification kit (Direct-zol RNA microprep, Zymo Research, California, USA). Complementary DNA (cDNA) was synthesized with a Reverse Transcription system (iScript supermix, BioRad, California, USA) according to the manufacturer’s protocol. Quantitative polymerase chain reaction (PCR) was performed with the TaqMan Gene Expression Assay probes (Table S7) (Thermo Fisher Scientific). *Gapdh* was used as the internal control to normalize the sample differences. Relative expression was calculated using the ΔΔCt values, and results were expressed as 2^−ΔΔCt^.

### Measurement of glycosaminoglycan release from cartilage explants

Femoral head cartilages (femoral cap) were harvested from 4-week-old SAMR1 and SAMP8 mice and weighed. The amount of the released glycosaminoglycan into the conditioned medium was measured using the Blyscan Glycosaminoglycan assay kit (Biocolor, UK) as previously described [17]. After normalized by femoral caps’ weight, the data are expressed as fold differences compared with the amount of glycosaminoglycan into the culture medium with the average of from control SAMR1 strain set to 1.

### Statistical analysis

In a pilot study, our comparison of the 23-week-old OARSI score between the 23-week-old SAMR1 group (*n* = 5) and the SAMP8 group (*n* = 5) for checking sample size showed an effect size of 2.5 by power analysis using G*power (version 3.1.9.2) (SAMR1: 1.62 ± 0.12, SAMP8: 7.54 ± 3.32). Using this effect size, we calculated the sample size (the power of 0.8 and a significance level of 0.05) and found that at least 6 animals were required in each group. To account for potential dropouts and increase power, the number of animals was increased to 10 in each group (only 23-week-old mice are *n* = 20 in each group). Thus, we evaluated SAMR1 and SAMP8 as follows SAMR1 *n* = 10; SAMP8 *n* = 10 at 6, 9, 11, 14, 18, 28, and 33 weeks of age. For the 23-week time point, we allocated 20 mice per strain to establish as reasonable point for OA evaluation because SAMP8 reliably exhibited OA-like pathologic changes in all joint tissues (cartilage, subchondral bone, meniscus, and synovium). Actual measurement value data are presented as mean ± standard deviation (S.D). Comparison of mean values was performed using Welch’s *t* test. The *p*-values were corrected using the Holm-Sidak method for multiple comparison (GraphPad Prism9). Data of body weight was analyzed by two-way factorial ANOVA. Scoring data were compared between SAMR1 vs SAMP8 in aging analysis with Mann–Whitney *U* test at each time point. Comparison of right and left knees of SAMP8 mice were performed using the Wilcoxon signed-rank test at each time point. The *p*-values were corrected by using Holm’s method for multiple comparison correction (BellCurve for Excel,ver.3.23). Kendall’s tau-b partial correlation coefficient was used to examine the correlation between the data of OARSI, meniscus, synovial, and subchondral bone scoring. Differences were considered statistically significant at ^#^ = *P* < 0.05 and ^##^ = *P* < 0.01.

## Results

### Pathological changes in knee joint tissues with aging

**Fig. 1:**
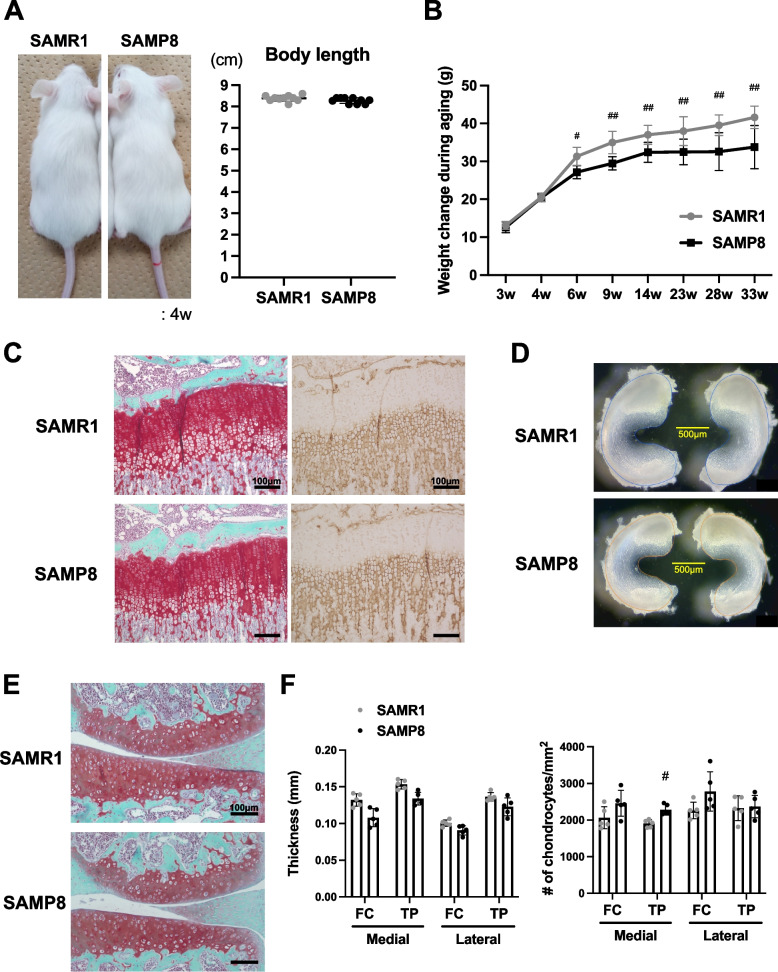
SAMP8 exhibit normal skeletal development and knee joint. **A** Full-body photographs of SAMR1 and SAMP8 were captured at 4 weeks of age (*n* = 10 for each strain). No significant difference in body length was observed. **B** Body weight of SAMP8 was changed with aging from 3 to 33 weeks of age (*n* = 10 for each strain). Data of body weight was analyzed by two-way factorial ANOVA. **C** Safranin O/Fast Green staining and type X Collagen staining in growth plate at 4 weeks of age. **D** Menisci structural images were obtained from SAMR1 and SAMP8 at 4 weeks of age. **E, F** Cellularity in articular cartilage surface at 4 weeks of age. Cartilage thickness of each part is the average thickness from horizontal quinque-section points over three different sections of one sample. Data represented as mean ± S.D. Comparison of mean values was performed using Welch’s *t* test. ^#^*P* < 0.05, ^##^*P* < 0.01 versus SAMR1. FC: Femoral condyle, TP: Tibial plateau

SAMP8 and SAMR1 at 4 weeks of age showed no difference in overall appearance. However, the body weight of SAMP8 was significantly lower than in SAMR1 from 6 weeks of age (Fig. 1A, B). Abnormalities in neither the cellularity of the growth plate (proliferative zone and type X Collagen positive-hypertrophic zone) and articulate cartilage nor the structure and shape of the knee joints and their menisci were observed in SAMP8 at 4 weeks of age (Fig. 1C–F). However, the density of chondrocytes was significantly higher in the medial tibial plateau of SAMP8 than that of SAMR1 (Fig. 1F). These findings indicate normal joint development, postnatal growth, and maturation in SAMP8. No mice died by 33 weeks of age in this study.

**Fig. 2:**
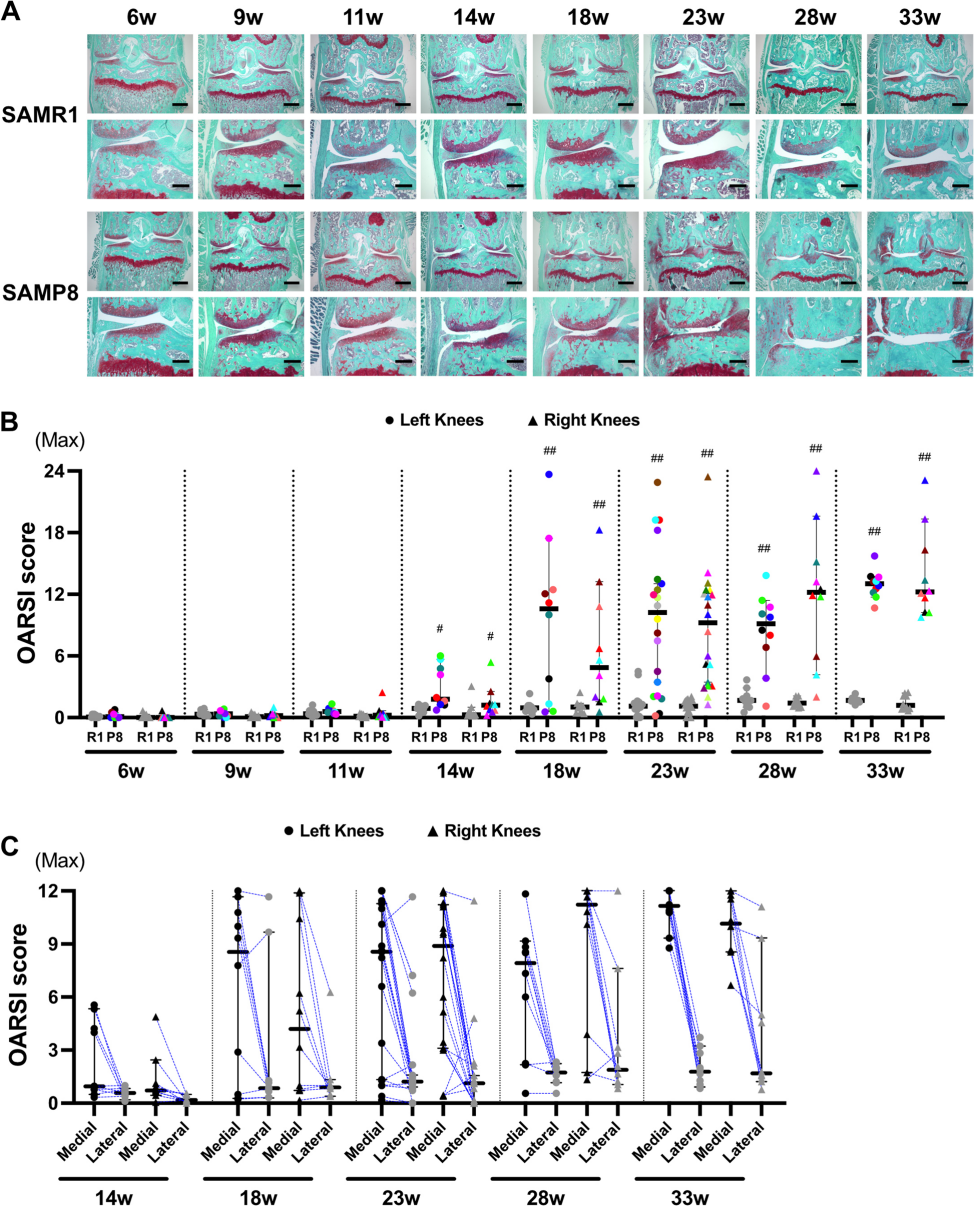
SAMP8 exhibit spontaneous osteoarthritis-like pathology in knee cartilage with aging. **A** Safranin O staining of the knee joint in SAMR1 and SAMP8 at 6 to 33 weeks of age (SAMR1, *n* = 10; SAMP8, *n* = 10 at 6, 9, 11, 14, 18, 28, 33 weeks and SAMR1, *n* = 20; SAMP8, *n* = 20 at 23 weeks). **B** Histopathologic scores (individual scores of left and right knee joints in SAMR1 and SAMP8 at 6 to 33 weeks of age). OARSI scores of entire knee joint were significantly increased in SAMP8 from 14 weeks of age. **C** OARSI scores of medial and lateral tibial/femoral cartilage of the left and right knee in the individual mice at 14 to 23 weeks of age in SAMP8. Round dots represent left knees and triangle dots represent right knees. The colors differentiate the individual mice in each time point. Scoring data represented as median with 95% confidence interval and were compared between SAMR1 and SAMP8 in aging analysis by Mann–Whitney *U* test at each time point. Comparison of right and left knees of SAMP8 mice were performed using Wilcoxon signed-rank test at each time point. The *p*-values (^#^*P* < 0.05, ^##^*P* < 0.01) were corrected using the Holm’s method for multiple comparison correction

To investigate whether SAMP8 exhibit an early onset of OA-like changes in cartilage, both knee joints from SAMP8 and SAMR1 were evaluated starting at 6 weeks of age. From 6 to 9 weeks of age, SAMP8 and SAMR1 showed intact articular cartilage and similar proteoglycan staining (Fig. 2A). Starting at 11 weeks of age, SAMP8 mice showed reduced Safranin O staining especially in the medial tibial plateau, indicating proteoglycan loss, a roughened articular surface, and fibrillations that were not associated with synovial hyperplasia, compared with normal tissue appearance in SAMR1 at the same age. Medial menisci degeneration and chondrophyte formation at the margin of the medial tibia was observed in SAMP8. At 18 to 33 weeks of age, SAMP8 exhibited partial or full-thickness cartilage defects with exposure of subchondral bone on the medial tibial plateau with menisci degeneration, synovial hyperplasia and osteophytes in all cases (Fig. 2A). None of these changes were observed in age-matched SAMR1 (Fig. 2A). The individual OARSI scores of all knee joints (left and right knee joints) indicated a significant increase in OA severity compared with SAMR1 at 14 weeks (median (interquartile range), SAMR1 vs SAMP8) [Left: 0.89 (0.56–1.07) vs 1.78 (1.35–4.62), Right: 0.28 (0.24–0.53) vs 1.21 (0.74–1.36)], 18 weeks [Left: 0.94 (0.74–1.14) vs 10.58 (1.94–12.35), Right: 1.03 (0.46–1.21) vs 4.86 (1.88–9.81)], 23 weeks [Left: 1.11 (0.63–1.40) vs 10.24 (3.11–13.13), Right: 1.11 (0.67–1.67) vs 9.22 (3.36–12.15)], 28 weeks [Left: 1.67 (1.20–2.04) vs 9.14 (7.13–10.58), Right: 1.41 (1.15–1.76) vs 12.19 (7.42–14.68)], and 33 weeks [Left: 1.67 (1.61–1.81) vs 13.03 (12.26–13.57), Right: 1.19 (0.89–2.11) vs 12.22 (10.62–15.60)] (Fig. 2A, B, Table S1).

**Fig. 3:**
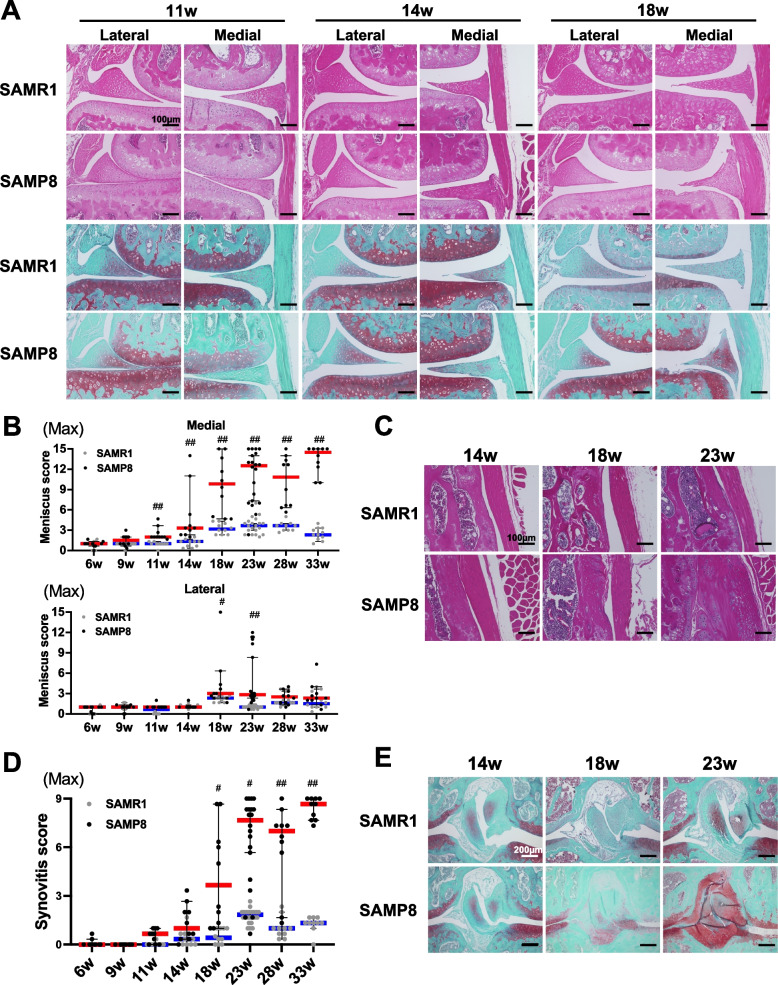
Aging-associated pathological changes in meniscus, synovium, and cruciate ligament. **A** H&E and safranin O staining of the medial meniscus in SAMR1 and SAMP8 at 11 and 14 weeks of age. **B** Histopathologic meniscus scores in medial and lateral knee menisci of left knee joints in SAMR1 and SAMP8 at 6 to 33 weeks of age. **C** H&E staining of the synovium in SAMR1 and SAMP8 at 14, 18, and 23 weeks of age. **D** Histopathologic scores in synovium in SAMR1 and SAMP8 at 6 to 33 weeks of age. **E** Safranin O staining of the cruciate ligament in SAMR1 and SAMP8 at 14, 18, and 23 weeks of age. All data are represented as median with 95% confidence interval were compared between SAMR1 and SAMP8 in aging analysis by Mann–Whitney *U* test at each time point. The *p*-values (^#^*P* < 0.05, ^##^*P* < 0.01) were corrected by using Holm’s method for multiple comparison correction

There was no significant difference in OARSI scores between left and right knee joints in SAMP8 at any time point (Fig. 2B and Table S6). OA severity was markedly increased in the medial compartment compared with the lateral compartment (Fig. 2A, C). Among the SAMP8 mice, 0% had mild OA, 20.0% had moderate OA and 80.0% had severe OA at 23 weeks of age in at least one knee joint (Fig. S1A). Among the knee joints (40 knee joints) in all SAMP8 at 23 weeks of age, 22.5% had moderate OA and 65.0% had severe OA (Fig. S1B). In addition, 45.0% of the mice had severe OA in both knee joints at 23 weeks of age (data not shown). Thus, SAMP8 exhibited OA-like structural changes in knee articular cartilage at 23 weeks of age. With advancing age (23–33-week-old), this increased up to 87.5% of the knee joints having severe OA (Fig. S1C, D). While there was no overall statistically significant difference in OA severity between left and right knees, there was mild asymmetry in 5/10 mice at 14 weeks, 3/10 at 18 weeks, 7/20 at 23 weeks, 3/10 at 28 weeks, and 0/10 at 33 weeks of age (Fig. 2B). We next investigated whether pathological changes in other knee joint tissues developed before the onset of cartilage damage in SAMP8 at 14 weeks of age. Analysis of menisci pathology by meniscus scoring of SAMP8 at 11 weeks of age indicated a significant increase in the severity of structural changes in medial meniscus, with surface fibrillations and undulations (Fig. 3A, B, and Table S2). Synovial score increased at 18 weeks of age subsequent to the onset of cartilage damage in SAMP8 at 14 weeks of age (Fig. 3C, D, and Table S3). Although cruciate ligaments were almost normal at 14 weeks of age, the histological changes such as rupture, mucoid degeneration, and chondrification, with joint inflammation were also seen after the onset of cartilage damage in SAMP8 at 14 weeks of age (Fig. 3E).

**Fig. 4:**
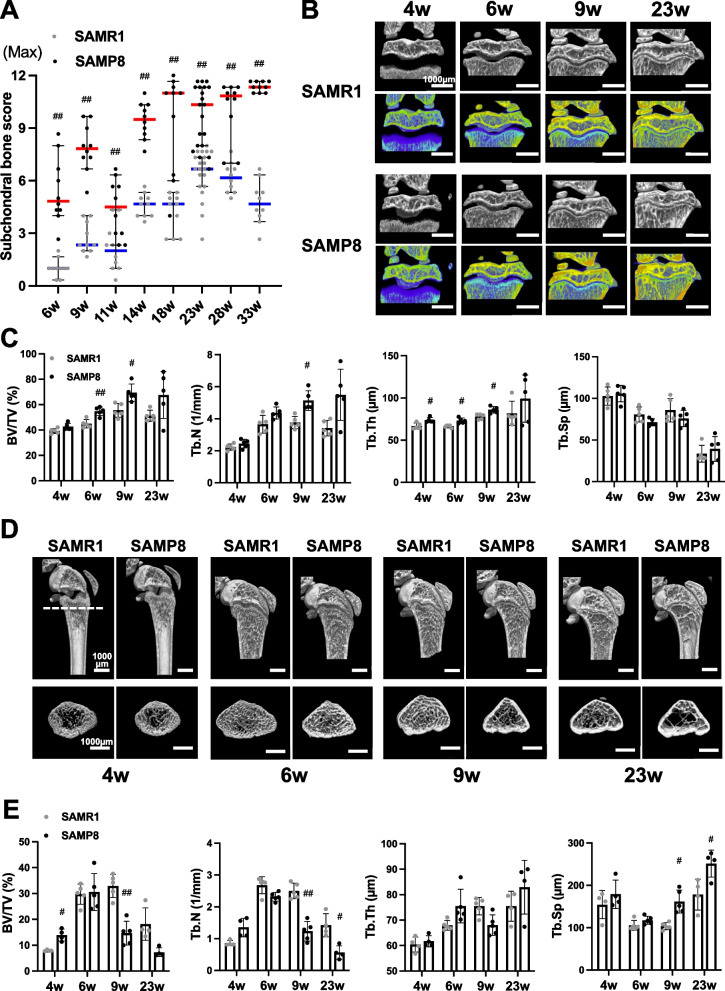
Analysis of subchondral area and femur metaphysis in SAMP8 with aging. **A** Histopathologic scores of subchondral bone in the medial tibia of SAMR1 and SMP8 at 4, 6, 9, 14, 18, 23, 28, and 33 weeks of age. Data are represented as median with 95% confidence interval were compared between SAMR1 and SAMP8 in aging analysis by Mann–Whitney *U* test at each time point. The *p*-values (^##^*P* < 0.01) were corrected by using the Holm’s method for multiple comparison correction. **B** Representative images of μCT and colored μCT with calcium phosphate from the same SAMP8 and SAMR1 at 4, 6, 9, and 23 weeks of age (*n* = 5 for each strain). **C** Graphs representing the parameters (bone volume (BV/TV), Tb.N (1/mm), trabecular thickness (Tb.Th, μm), and trabecular separation (Tb.Sp, μm)) of subchondral bone region of the medial tibia on μCT analysis. **D** μCT images of the metaphysis of the distal femur in SAMP8 and SAMR1 at 4, 6, 9, and 23 weeks of age (*n* = 4–5 for each strain). **E** Graphs representing the parameters of the metaphyseal region on μCT analysis. Data are represented as mean ± S.D. Comparison of mean values was performed using Welch’s *t* test. The *p*-values (^#^*P* < 0.05, ^##^*P* < 0.01) were corrected using the Holm-Sidak correction

SAMP8 at 6 weeks of age already showed sclerotic changes with decreased bone marrow cavities in the medial tibial subchondral bone and complete replacement of the bone marrow (BM) at 9 weeks of age (Fig. 2A). Subchondral bone scores using our recently established scoring system, which were at the same level at 4 weeks [13] showed significant differences in medial tibia between SAMP8 and SAMR1 at 6 to 33 weeks (Fig. 4A, Table S4). The mice evaluated with µCT are the same mice (4-, 6-, 9-week-old) from those previously reported [13]. We performed additional µCT analysis in the epiphysis of the medial tibias and the metaphysis of the distal femur. Indeed, calcium accumulation and BMD (BV/TV, %), trabecular number (Tb.N, 1/mm), and trabecular thickness (Tb.Th, μm) in the medial tibial plateau were significantly higher in SAMP8 compared to SAMR1 with aging (Fig. 4B, C). On the other hand, in the femoral metaphysis, BMD, and trabecular number were significantly decreased in SAMP8 compared with SAMR1 from 9 weeks of age (Fig. 4D, E). Trabecular separation (Tb.Sp, μm) was significantly increased in SAMP8 compared with SAMR1 from 9 weeks of age (Fig. 4E). These results indicated that sclerosis was increased in the epiphysis of the medial tibias, while bone density was decreased in the metaphysis of the distal femur in SAMP8.

### OA-related markers in blood and articular cartilage of SAMP8

To examine inflammatory condition in SAMP8, inflammatory cytokine in serum was measured. However, IL-6 levels were low in both SAMR1 and SAMP8 (mean values 50.8 pg/ml and 40.0 pg/ml, respectively) at 9 weeks of age before the onset of cartilage damage (data not shown).

**Fig. 5:**
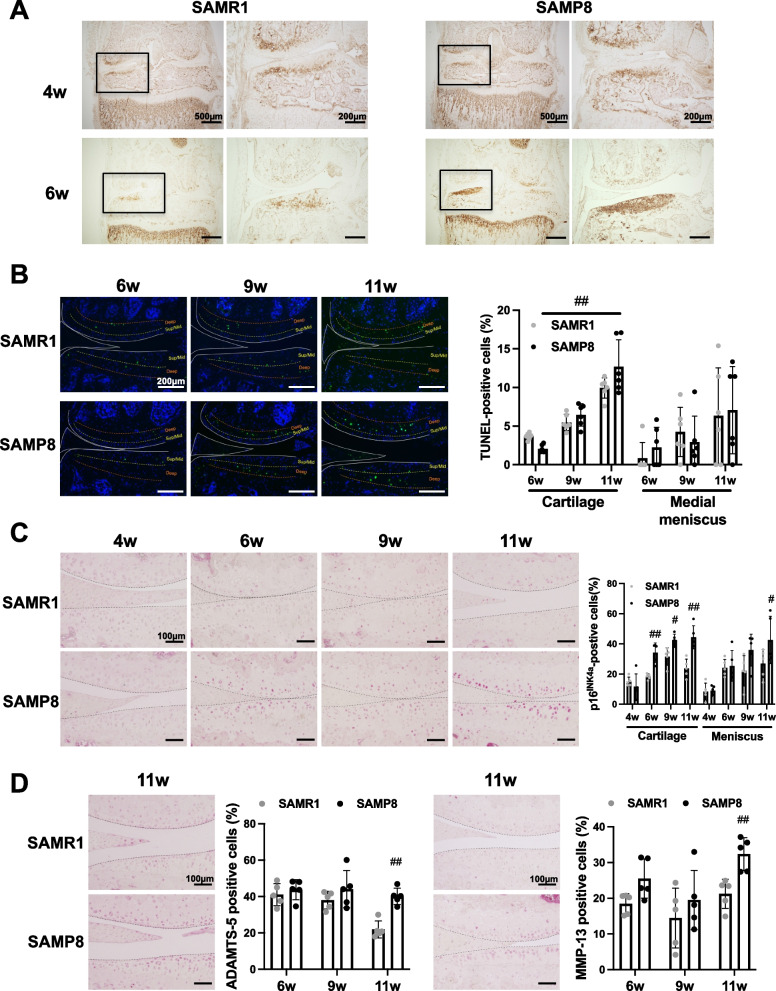
Osteoarthritis related markers in the articular cartilage of SAMP8. **A** The expression pattern of type X Collagen in articular cartilage of SAMR1 and SAMP8 at 4 and 6 weeks of age. Knee joints were assessed by immunohistochemistry. **B** Medial knee joints from SAMR1 and SAMP8 at 6, 9, and 11 weeks of age (*n* = 6 for each strain) were assessed by TUNEL staining. White lines: outline of articular cartilage/meniscus; Dashed lines show borders between middle/deep zone or deep zone/subchondral bone. **C** Knee joints from SAMR1 and SAMP8 at 4, 6, 9, and 11 weeks of age (*n* = 5 for each strain) were assessed by immunohistochemistry using anti-p16^INK4a^ antibody.** D** Immunohistochemistry using anti-ADAMTS-5 and anti-MMP-13 antibodies in medial compartment of the knee from SAMR1 and SAMP8 at 6, 9, and 11 weeks of age (*n* = 5 for each strain). All data are represented as mean ± S.D. Comparison of mean values was performed using Welch’s *t* test. The *p*-values (^#^*P* < 0.05, ^##^*P* < 0.01) were corrected using the Holm-Sidak method for multiple comparison correction

We focused on the events in articular cartilage that occur at 4 to 11 weeks of age before the onset of macroscopic and histological cartilage degradation. Chondrocyte hypertrophy in articular cartilage has been characterized as a pathological change of OA and inhibiting chondrocyte hypertrophy has been considered as a target for OA treatment [18–20]. The hypertrophic chondrocyte marker type X Collagen was widely expressed from the deep layer to the surface layer of articular cartilage on the medial tibia in SAMP8 from 6 weeks of age (Fig. 5A). Type X Collagen in SAMR1 was only expressed in the deep layer of articular cartilage (Fig. 5A). However, the expression pattern of type II Collagen in SAMP8 was similar with SAMR1 from 4 to 14 weeks of age (Fig. S2A).

**Fig. 6:**
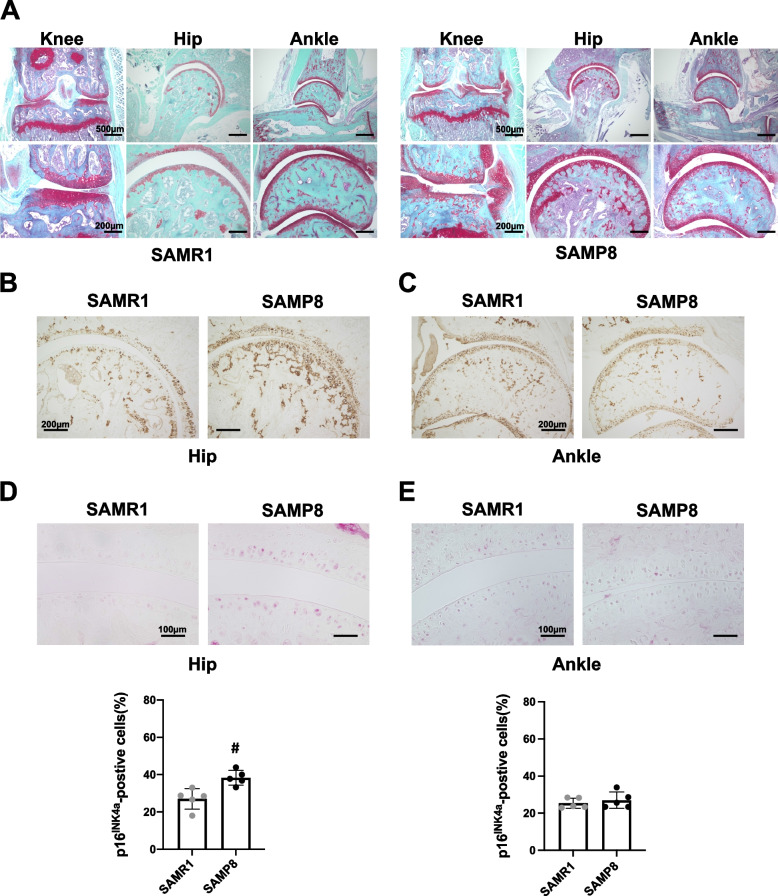
Pathological changes in ankle and hip joints in SAMP8 with aging. **A** Safranin O staining of the knee, hip and ankle joints in SAMR1 and SAMP8 at 23 weeks of age. **B, C** Immunohistochemistry using anti-type X Collagen on hip and ankle joints from SAMR1 and SAMP8 at 23 weeks of age. **D, E** Immunohistochemistry using anti-p16^INK4a^ antibodies on hip and ankle joints from SAMR1 and SAMP8 at 23 weeks of age (ankle and hip: *n* = 5 for each strain). All data represented as mean ± S.D. Comparison of mean values was performed using the Welch’s *t* test; ^#^*P* < 0.05 versus SAMR1

Chondrocyte apoptosis and senescence have been detected in OA cartilage and play an important role in OA pathogenesis [21–23]. From 6 to 11 weeks of age before the onset of cartilage damage, TUNEL-positive cells were increased with age and were mainly localized in the middle to deep zone of medial articular cartilage (Fig. 5B). However, there were no significant differences in articular cartilage and menisci between SAMR1 and SAMP8 (Fig. 5B). The numbers of cells positive for p16^INK4a^, a marker of senescent or dysfunctional chondrocytes [22–26], were significantly increased in articular cartilage of SAMP8 from 6 weeks of age. In menisci, there were significant differences at 11 weeks of age (Fig. 5C). ADAMTS-5 and MMP-13, which are key enzymes in cartilage degradation, were also significantly increased in articular cartilage of SAMP8 compared with SAMR1 at 11 weeks of age (Fig. 5D). Cells expressing these markers were significantly abundant in the medial side than in the lateral side, consistent with the severity of OA (Fig. S4). We also examined the hip and ankle joints in SAMR1 and SAMP8. Although SAMP8 exhibited severe OA in knee joints at 23 weeks of age, cartilage degradation in hip and ankle joint of SAMP8 was not exhibited (Fig. 6A). There was thus no association between knee OA and OA in hip or/and ankle joints in SAMP8 (Fig. 6A). However, cartilage degradation was observed in the tarsometatarsal joint in both SAMP8 and SAMR1 at 23 weeks of age (Fig. S3A). Furthermore, although type X Collagen and p16^INK4a^-positive senescent cells were detected, SAMP8 did not exhibit severe OA in hip and ankle joints at 23 weeks (Fig. 6B–E) and 52 weeks of age (Fig. S3B).

### Gene expression in knee articular cartilage of SAMP8

**Fig. 7:**
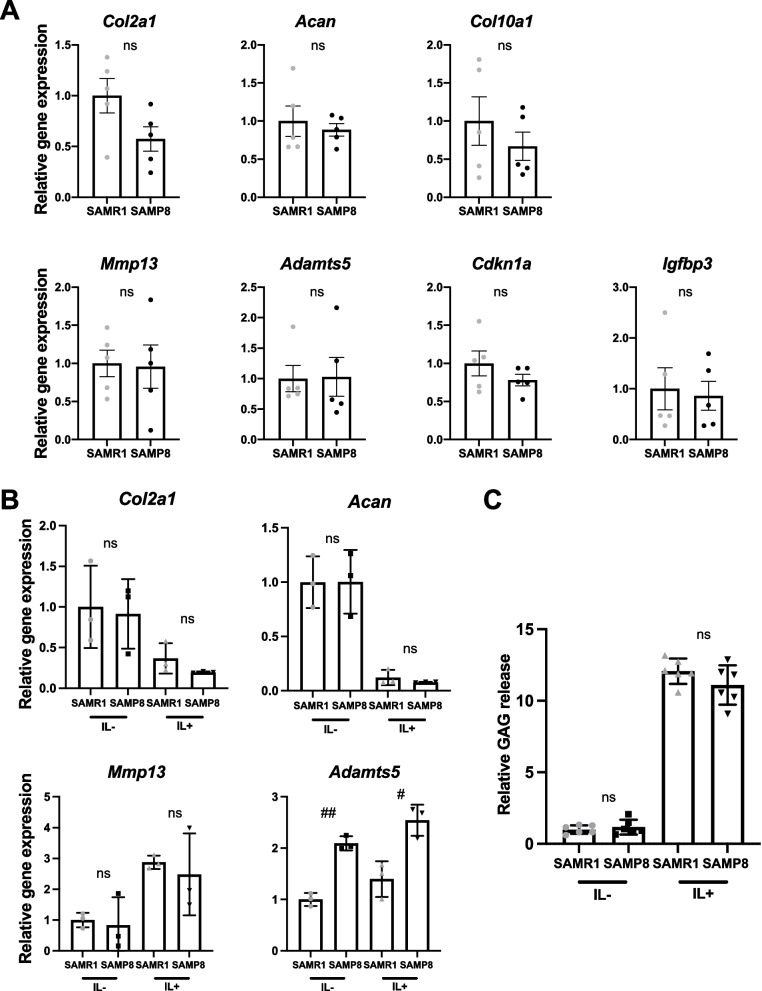
Gene expression of articular cartilage tissue and cultured chondrocytes in SAMP8. **A** The expression of OA-related genes in articular cartilage tissues from knee joint of SAMR1 and SAMP8 (*n* = 5 for each strain) at 4 weeks of age. **B** Articular chondrocytes were isolated from the femoral heads of SAMR1 and SAMP8 at 4 weeks of age. The expression of OA-related genes in SAMR1 and SAMP8 chondrocytes with or without IL-1β (1 ng/ml) for 24 h (*n* = 3 each group). **C** Femoral head cartilage explants from SAMR1 and SAMP8 (*n* = 6 each group) cultured with or without IL-1β (5 ng/ml) for 72 h. Proteoglycan release into the conditioned medium from cartilage was assayed as the concentration of glycosaminoglycan (GAG) and presented as fold changes to the average of SAMR1(IL-1). All data represented as mean ± S.D. Comparison of mean values was performed using Welch’s *t* test. The *p*-values (^#^*P* < 0.05, ^##^*P* < 0.01) were corrected using the Holm-Sidak method for multiple comparison correction. ns: not significant

To determine whether cells in cartilage of SAMP8 mice have intrinsic changes or defects that lead to early-onset OA, we analyzed gene expression patterns and in vitro functions of chondrocytes and articular cartilage from mice at 4 weeks when there are no OA-like histological changes detectable in cartilage. The expression of OA- or senescence-related genes in articular cartilage tissue was not significantly different between SAMR1 and SAMP8 (Fig. 7A). *Cdkn2a* and senescence-associated secretory phenotype (SASP) factor IL-6 were undetected in articular cartilage. Cultured articular chondrocytes showed that basal levels or IL-1β induced changes of *Adamts5* was significantly increased in SAMP8, while *Col2a1*, *Acan*, and *Mmp13* were not different between SAMR1 and SAMP8 (Fig. 7B). To further evaluate the phenotype of articular chondrocytes from SAMP8, we quantified glycosaminoglycan release from mouse femoral head cartilage explants from SAMR1 and SAMP8 at 4 weeks of age. IL-1β significantly increased proteoglycan release, but there was no significant difference between SAMR1 and SAMP8 (Fig. 7C).

### Relationship of knee joint tissue changes in SAMP8

Finally, we analyzed the correlation of pathological changes between joint tissues (articular cartilage, meniscus, synovium, and subchondral bone) of 14–33-week-old SAMP8 mice (Table S5). There were correlations between synovitis and cartilage damage (*r* = 0.8827, *p* < 0.0001), meniscus degeneration and cartilage damage in medial compartment (*r* = 0.8310, *p* < 0.0001), and subchondral bone sclerosis and cartilage damage (*r* = 0.5702, *p* < 0.0001). These results indicated that the cartilage damage in SAMP8 is associated with the pathologic changes of knee joint tissues as typical OA pathology.

## Discussion

OA, an aging-related disease, develops slowly over a long period of time. Thus, in most mouse strains, OA-like structural changes with severe cartilage damage are slow to progress and are not detectable until around 15–22 months of age [3–6]. In addition, there are variations in the incidence and severity of OA within and among mouse strains [2, 27, 28]. Although the STR/Ort mouse is the most widely used model of spontaneous OA and has been featured in over 80 studies [8], the present study shows that in SAMP8 mice, OA development is less variable, and progression is more rapid so that experiments using SAMP8 can be performed on a compressed timescale. Therefore, male SAMP8 is a mouse OA model that is useful for investigating the pathogenesis of primary OA as whole joint disease and for evaluating therapeutic interventions.

**Fig. 8:**
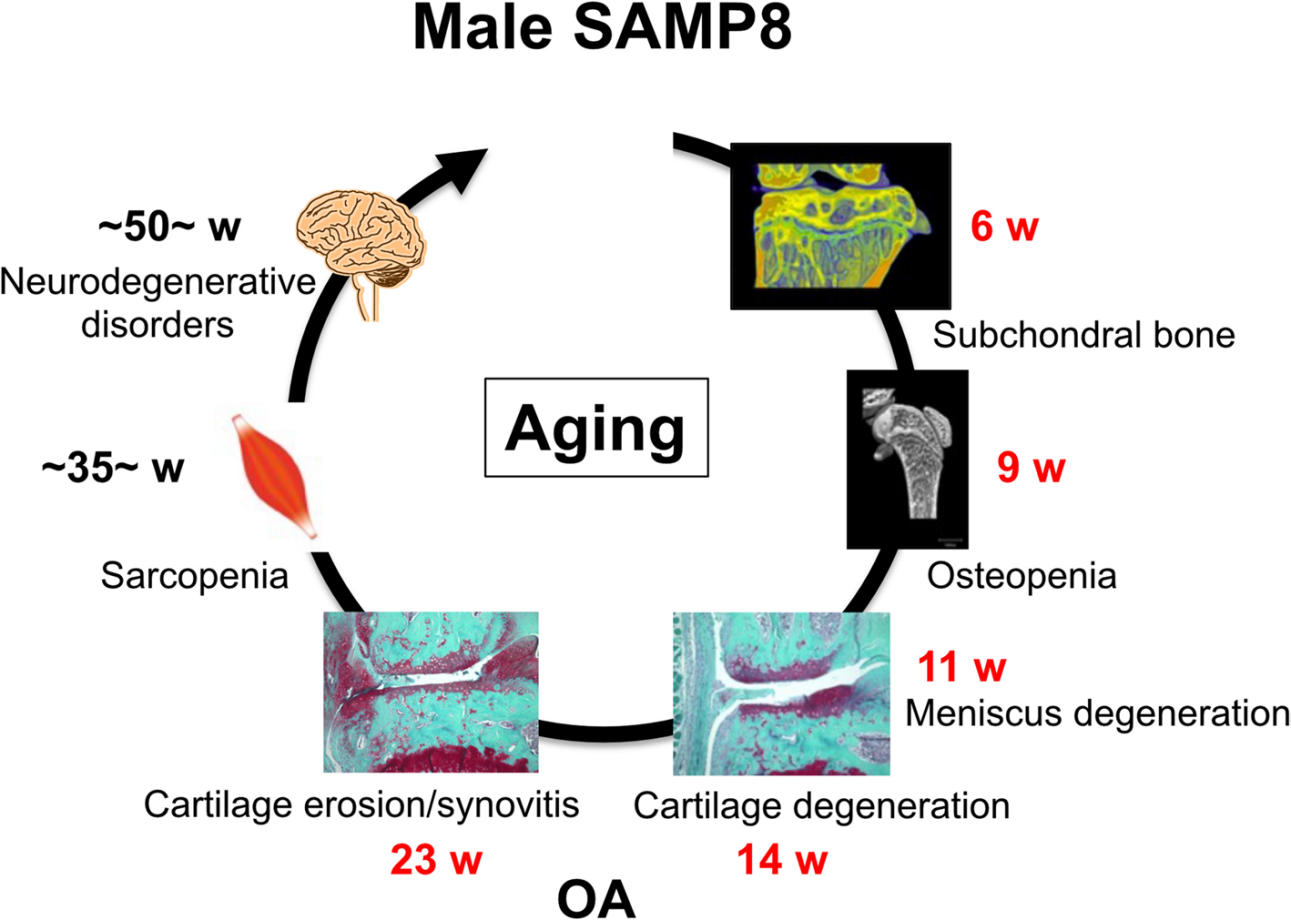
Major pathological changes in SAMP8 with aging. Schematic diagram describes main pathological changes in SAMP8 with aging. W: weeks of age

Although the reasons why SAMP8 display accelerated aging and various disease are still unknown, different combinations of mutations in disease-causing genes may be responsible for the various phenotypes of SAMP strains [29]. SAMP8 develops various aging-related phenotypes such as disruption of autonomic nervous function and circadian rhythm [30–32], neurodegenerative disorders [10], and sarcopenia [11]. However, osteopenia (9-week-old) and OA (14-week-old) occurred much earlier than the development of those diseases. Thus, clarifying the relationship between systemic changes originating from the changes of bone-cartilage may open new insight in aging-related diseases (Fig. 8, Table S5, S6).

Chondrocyte hypertrophy and senescent cells increased at 6 weeks before the onset of cartilage degradation at 14 weeks of age. Increased numbers of hypertrophic chondrocytes in articular cartilage have been observed in OA development, and inhibition of hypertrophy has been shown to reduce the severity of OA [18–20]. Age-related diseases have been characterized by the accumulation of senescent cells in various tissues including cartilage and are associated with age-related pathogenesis [24, 33–36]. According to another study, p16^INK4a^-positive senescent cells increase in articular cartilage with aging, but p16^INK4a^-positive cells are not essential for SASP production and are not involved in the development of OA [26]. Although p16^INK4a^ is recognized as senescent cell marker, relation between accelerated-aging phenotype and p16^INK4a^-positive senescent cells in SAMP8 has not been characterized yet. In the present study, although many chondrocytes in SAMP8 at 4 weeks of age did not express the senescence marker p16^INK4a^, the rate of p16^INK4a^-positive chondrocytes in SAMP8 was increased with aging than in SAMR1. However, p16^INK4a^ was also detected in chondrocytes of the lateral tibial plateau, ankle, and hip joint of SAMP8 which showed less cartilage degeneration than the medial compartment of knee joints at 23 weeks of age. Furthermore, ankle and hip joints expressed p16^INK4a^ but did not exhibit OA-like cartilage defects even at 52 weeks of age (Fig. S3A). Thus, abnormal expression of type X Collagen and p16^INK4a^-positive chondrocytes in the articular cartilage might not be an essential trigger in the development and progression of OA in SAMP8.

OA is whole joint disease [37], and lesion formation and degeneration of the menisci and cruciate ligaments contribute to the development and progression of OA [38, 39]. In our study, pathological changes of the meniscus in SAMP8 were observed before cartilage degeneration, whereas pathological changes of the cruciate ligament and synovium were observed in knee joints with cartilage degeneration after 14 weeks of age. Thus, the degeneration of cruciate ligaments, and synovitis, are subsequent to cartilage damage and these are likely not to be the major initiator of OA in SAMP8. Subchondral bone sclerosis and BMD loss of femoral metaphysis in SAMP8 were pathological changes that occur at 6 to 9 weeks before the onset of cartilage degradation at 14 weeks of age. The subchondral bone and articular cartilage act as a functional unit in the joint. Subchondral bone sclerosis in OA joints, although often considered secondary, occurs at an early phase of the OA process, and is closely associated with changes to the cartilage [37, 40–42]. Moreover, various subchondral bone-targeting therapeutic agents such as bisphosphonates have potential in OA treatment [40, 43]. Subchondral bone sclerosis in the medial tibia and BMD loss in the metaphysis of the distal femur should be investigated as targets for OA treatment. The functions of BM including bone remodeling and BMSCs in SAMP8 might be reduced with aging. Indeed, cultured BMSCs which were isolated from BM of SAMP8 at 6 months of age were positive for the senescence marker p16^INK4a^ [12]. Although the increasing adiposity and inflammatory cytokines in BM with aging are associated with abnormal bone remodeling [44], adiposity formation was not observed in BM of SAMP8 at 6 to 14 weeks of age. Transplantation of normal young BM into SAMP6 can normalize the BM microenvironment, with the imbalance between bone formation and resorption observed in the osteoporosis-prone SAMP6 mice being ameliorated [45, 46]. However, it is still not understood whether resident skeletal stem cells can contribute to maintenance of articular cartilage [47]. Thus, it might be interesting to determine whether BM transplants can rescue the development of OA in SAMP8. Primary abnormalities in subchondral bone may be a trigger for OA in SAMP8. However, we should further examine the causes of mechanical stress, because we have not reached a conclusion as to why only the knee joint and the medial compartment shows accelerated subchondral bone change and cartilage degeneration. Further elucidating whether the sequential pathological changes in different joint tissues are inter-connected or independent is important for OA pathogenesis. C57BL6/J mice develop OA earlier and more severe in male compared with female mice [48]. Although the present study evaluated male SAMP8 only, we should further examine whether the severity of OA in SAMP8 depends on sex. In addition, the locomotor activity of SAMP8 was similar to that of SAMR1 at 20 weeks of age [31]. The relationship between OA severity and pain should be evaluated by combination of various new tools such as gait analysis based on motion capture system using marker less using AI technology [49], although it is difficult to evaluate pain in mouse OA model.

Together, our study indicates that SAMP8 is a spontaneous and early-onset OA mouse model that is useful for evaluating OA pathogenesis. SAMP8 exhibited severe OA with the pathologic changes of knee joint tissues. A better understanding of the genes, molecules, and processes involved in the OA pathogenesis of SAMP8 will therefore contribute significantly to the identification of new preventative, protective, and therapeutic approaches for primary OA.

## Supplementary Information

**Additional file 1:** **Fig. S1.** Incidence of aging-related spontaneous osteoarthritis development in knees of SAMP8 at 23 weeks of age.

**Additional file 2:** **Fig. S2.** Histology of whole knee joint in SAMR1 and SAMP8.

**Additional file 3:** **Fig. S3.** Hip and ankle joints of SAMP8 with aging.

**Additional file 4:** **Fig. S4.** ADAMTS-5 and MMP-13-positive in medial and lateral compartment of the knee from SAMR1 and SAMP8.

**Additional file 5:** **Fig. S5.** Isotype control Images of all Antibodies used in Immunohistochemistry.

**Additional file 6:** Supplementary materials and methods.

**Additional file 7:** **Table S1.** OARSI score.

**Additional file 8:** **Table S2.** Meniscus score.

**Additional file 9:** **Table S3.** Synovitis score.

**Additional file 10:** **Table S4.** Subchondral bone score.

**Additional file 11: Table S5.**

**Additional file 12: Table S6.**

**Additional file 13: Table S7.**

## Data Availability

The datasets used and/or analyzed during the current study are available from the corresponding author on request.

## Abbreviations

OA: Osteoarthritis
SAMP8: Senescence-accelerated mouse prone 8
SAMR1: Senescence-accelerated mouse-resistant 1
BMD: Bone mineral density
DAPI: 4’,6-Diamidino-2-phenylindole
BM: Bone marrow
SASP: Senescence-associated secretory phenotype
