# Supplementary material for: Senescence-accelerated mice prone 8 (SAMP8) in male as a spontaneous osteoarthritis model
Source: Arthritis Res Ther. 2022 Oct 18;24:235. doi: 10.1186/s13075-022-02916-5 (PMC9578281; doi:10.1186/s13075-022-02916-5)
Supplement: Supplementary file 6 — Additional file 6: Supplementary materials and methods. [file 13075_2022_2916_MOESM6_ESM.docx]

**SUPPORTING INFORMATION**

**Immunohistochemical analysis**

Knee joint sections were deparaffinized with xylene and a descending ethanol series before rehydration. Slides were pretreated with antigen retrieval reagent (Proteinase K, Dako) at room temperature for 10min, followed by normal blocking serum for 30 minutes. Sections were immunostained with anti-Type II collagen (DSHB, CIIC1, 5.6µg/mL) diluted in PBS, using VECTASTAIN Elite ABC-HRP kit and DAB substrate kit (Vector Laboratories, Burlingame, CA, USA) according to the manufacturer’s instructions.
